# Impact of some inorganic anions on the corrosion of nickel in a solution containing Na_2_SO_4_ and NaClO_4_

**DOI:** 10.1038/s41598-024-52281-3

**Published:** 2024-01-22

**Authors:** M. A. Deyab, Majed M. Alghamdi, Adel A. El-Zahhar

**Affiliations:** 1 Egyptian Petroleum Research Institute, Nasr City, 11727 Cairo Egypt; 2 Department of Chemistry, college of Science, King Khalid University, P.O. Box 9004, 61413 Abha, Saudi Arabia

**Keywords:** Chemistry, Electrochemistry

## Abstract

Potentiodynamic study was carried out on nickel in Na_2_SO_4_ solution in the presence of ClO_4_^–^, WO_4_^2–^, MoO_4_^2–^, NO_2_^–^ and NO_3_^–^ ions. The anodic excursion spans of the metal nickel in a solution of Na_2_SO_4_ are marked by the appearance of clearly defined anodic peak, passive region, and transpassive shoulder. According to the data, the anodic peak current density (I_PAI_) rise from 1.82 to 8.12 mA cm^–2^ as the concentration of the Na_2_SO_4_ solution rises from 0.2 to 1.0 M. It is clear that as scan rate increases, the I_PAI_ rises reaching to 11.8 mA cm^–2^. The apparent activation energy of nickel corrosion in Na_2_SO_4_ is 33.25 kJ mol^–1^. ClO_4_^–^ anion addition speeds up nickel’s active dissolution, as well tends to break down the passive layer, and causes pitting penetration. It was found that, the pitting potential (E_pit_) of nickel in solutions containing the two anions ClO_4_^–^ and SO_4_^2–^ shifts to the positive direction by addition of WO_4_^2–^, MoO_4_^2–^, NO_2_^–^ anions and shifts to the negative direction by addition NO_3_^-^ anion. E_pit_ increased by 0.67, 0.37 and 0.15 V in the presence of WO_4_^2–^, MoO_4_^2–^ and NO_2_^–^, respectively. WO_4_^2–^ > MoO_4_^2–^ > NO_2_^–^ was the order in which the inhibitors were most effective.

## Introduction

Nickel has attracted many investigations of its electrochemical and other properties in different electrolytes^1–4^, due to its wide speared industrial applications. Nickel is a metal with strong resistance to corrosion according to many circumstances; however, it may still corrode under some conditions. Nickel corrosion behavior is affected by environmental variables, including the existence of corrosive chemicals, and the specific type of nickel (for instance, native nickel, nickel alloys).

Nickel may corrode in corrosive conditions, especially those including halides e.g. chlorides. The incorporation of halides can hasten the corrosion process by breakdown the passivity of the nickel surface^5–7^. The proposed mechanisms in which chlorides ions are adsorbed on the nickel surface in competition with water molecules as the following:

1 $${\text{Ni }} + {\text{ H}}_{{2}} {\text{O }} \to {\text{ Ni}}\left( {{\text{H}}_{{2}} {\text{O}}} \right)_{{{\text{ads}}}} ,$$

2 $${\text{Ni }} + {\text{ Cl}}^{ - } \to {\text{ Ni}}\left( {{\text{Cl}}^{ - } } \right)_{{{\text{ads}}}} ,$$

3 $${\text{Ni}}\left( {{\text{Cl}}^{ - } } \right)_{{{\text{ads}}}} \to {\text{ NiCl}} + {\text{ e}}^{ - } ,$$

4 $${\text{NiCl }} + {\text{ Cl}}^{ - } \to {\text{ NiCl}}_{{{2}({\text{aq}}.)}} + {\text{ e}}^{ - } .$$

In the presence of other different species, including perchlorate, sulphate, and nitrate ions, localized corrosion of nickel can also happen^8^. Several corrosion preventative methods can be used to reduce nickel corrosion that includes:

*Coatings*: Using protection coatings like nickel plating, electroless metal coatings, as well as organic coatings might give an extra shield towards corrosion^9^.

*Alloying*: To improve corrosion resistance in certain settings, the alloy of nickel with additional elements can be used. Because of their superior resistance to different corrosive chemicals, nickel-based alloys such as Inconel or Hastelloy alloy are frequently used in corrosive settings^10^.

*Cathodic* *protection*: Using cathodic protection methods including sacrificial electrodes system or impressed current structures, nickel may be protected from corrosion^11^.

Nickel corrosion resistance may be significantly improved by chromate ions^12,13^. Chromate is first adsorbed on the cathodic sites of the metal surface, followed by electrochemical cathodic reduction to give some kinds of Cr oxides. This oxide layer acts as a barrier from further corrosion by blocking corrosive substances such as oxygen and moisture from accessing the underlying metal. By encouraging the creation of a stable and protective chromium oxide layer, the chromate ion passivation technique can improve the corrosion resistance of nickel. In alkaline or somewhat acidic situations, chromate passivation is especially effective. But as a result to their toxicity, hexavalent chromium material, such as chromate ions have recently caused environmental issues. As a consequence, studies regarding new, environmentally conscious corrosion inhibitors and passivation approaches have changed.

In the current work, alternative inorganic materials, WO_4_^2–^, MoO_4_^2–^, NO_2_^–^, NO_3_^–^ ions are being explored for their anti-corrosion properties on nickel in Na_2_SO_4_ solution in the presence of perchlorate ions. These ions are considered as a less toxic alternative to hexavalent chromium compounds. Ongoing research aims to optimize the use of these inorganic materials and develop effective passivation materials for nickel and its alloys.

## Experimental

Polarization measurements were performed on cylindrical electrodes machined nickel rod (99.98%, surface area 0.463 cm^–2^). Every time, the nickel surface was cleaned up by using emery paper for polishing from no. 400 to no. 2500. Finally, the working electrode was washed with double distilled water and degreased with acetone. Studies on potentiodynamic polarization were conducted using a computer and a potentiostat instrument (Gamry-3000). The tests have been carried out in a standard three-compartment cell employing a saturated calomel electrode to be the reference electrode, a platinum foil to be the auxiliary electrode, and nickel to be the working electrode. The polarization curves were swept from − 2.0 to 2.0 V. Potential scan rate range from 20 to 100 mV s^–1^.

Chemicals of analytical grade and three-time distilled pure water were used to create solutions. For all studies, various concentrations of Na_2_SO_4_ solutions were prepared using laboratory-grade Na_2_SO_4_ (Merck) and triple distilled water. Sigma-Aldrich Company supplied the inorganic compounds (NaClO_4_, Na_2_WO_4_, NaNO_2_, NaNO_3_), which were utilized without any extra purification. By submerging the cell in a water thermostat, the temperature of the experiment’s solution was regulated with a temperature range of 30–70 °C. All experiments were conducted in aerated solutions.

The surface morphology of some samples after immersion for 120 h at 30 °C was performed using Scanning Electron Microscope (SEM) ZEISS Gemini-SEM Microscopy.

## Results and discussion

### Electrochemical polarization of nickel in Na_2_SO_4_ solution

**Figure 1:**
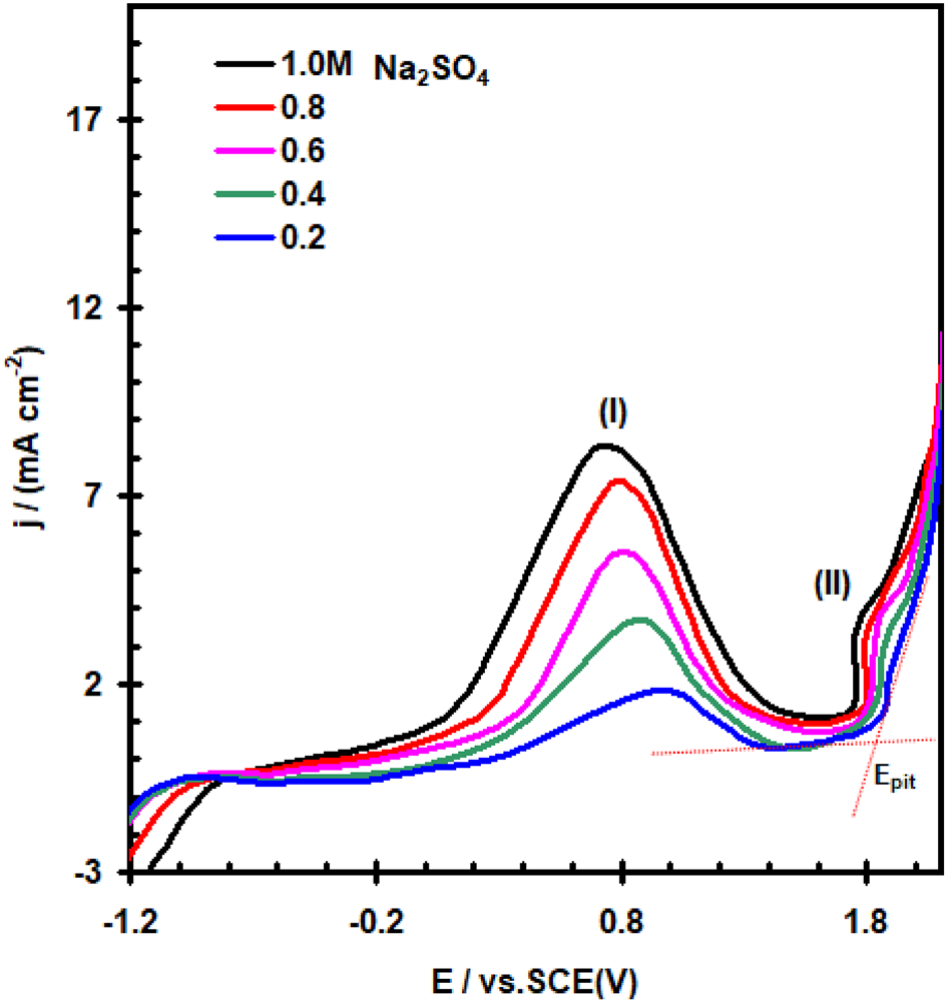
Polarization curve for nickel electrode in Na_2_SO_4_ solution at 30 °C with scan rate of 20 mVs^–1^.

Typical polarization curves of nickel in various concentrations of Na_2_SO_4_ solution (0.2 to 1.0 M) are given in Fig. 1. The curves were swept from -2.0 V to 2.0 V at scan rate υ = 20 mVs^–1^ and at 30 °C. On positive going scan, the cathodic current density decreases continually reaching corrosion potential (E_corr_) at zero current density. Prior to the oxygen evolution reaction, each anodic curve shows one dissolution peak (I), a definitive passive state, and a transpassive state (II). The dissolution peak (I) of nickel is related to electroformation of hydrous Ni(OH)_2_^14–16^ layer, which is transformed chemically and electrochemically to produce the passive NiO layer in the end^17–19^. When the concentration of nickel oxide exceeds the solubility product of NiO, precipitation of a porous oxide film occurs on the electrode surface. The current decreases to an extremely low value, signalling the beginning of primary passivation, when the surface is completely covered with a nonporous passive film. The passivation current start to increase markedly, forming transpassive state prior to oxygen evaluation. Sato and Kamoto^20–23^ attributed this behaviour to the dissolution of Nickel yielding Ni^2+^ ions, through active spots in the passive film.

The anodic peak current density (I_PA1_) rise from 1.82 to 8.12 mA cm^–2^ as the concentration of the Na_2_SO_4_ solution rises from 0.2 to 1.0 M, as shown in Fig. 1. Furthermore, as the concentration of the Na_2_SO_4_ solution increases, the anodic peak potential relatively shifts in a less positive direction.

**Figure 2:**
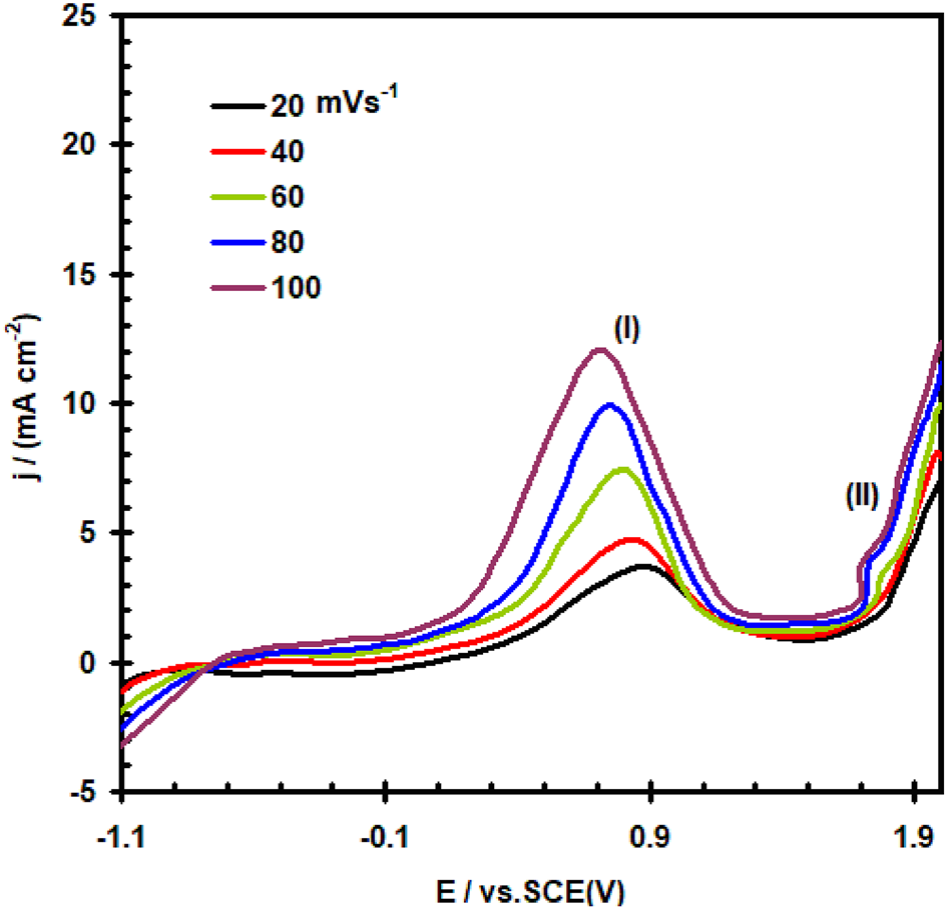
Polarization curve for nickel electrode in 0.4 M of Na_2_SO_4_ solution at 30 °C at different scan rate.

**Figure 3:**
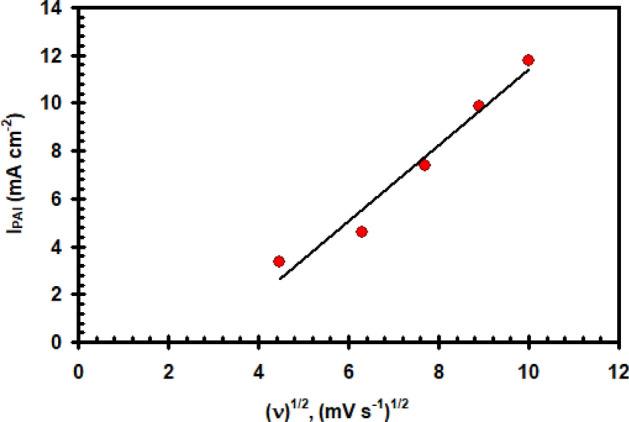
Relation between (I_PAI_) vs. the square root of scan rate for nickel electrode in 0.4 M of Na_2_SO_4_ solution at 30 °C.

Figure 2 depicts the findings of this study on the effect of potential scan rate (υ) on the potentiodynamic polarization of a nickel electrode in 0.4 M Na_2_SO_4_ solution at 30 °C. It is clear that as υ increases, the peak current density (I_PA1_) rises reaching to 11.8 mA cm^–2^ and its peak potential (E_PA1_) moves towards more negative values. Good linearity of the (I_PA1_) verses υ^1/2^ plot was observed (Fig. 3), but do not pass through the origin. It illustrates that a combined operation including direct film formation and diffusion-controlled dissolve may be used to explain the oxide layer formation process in the potential area of peak A1.

**Figure 4:**
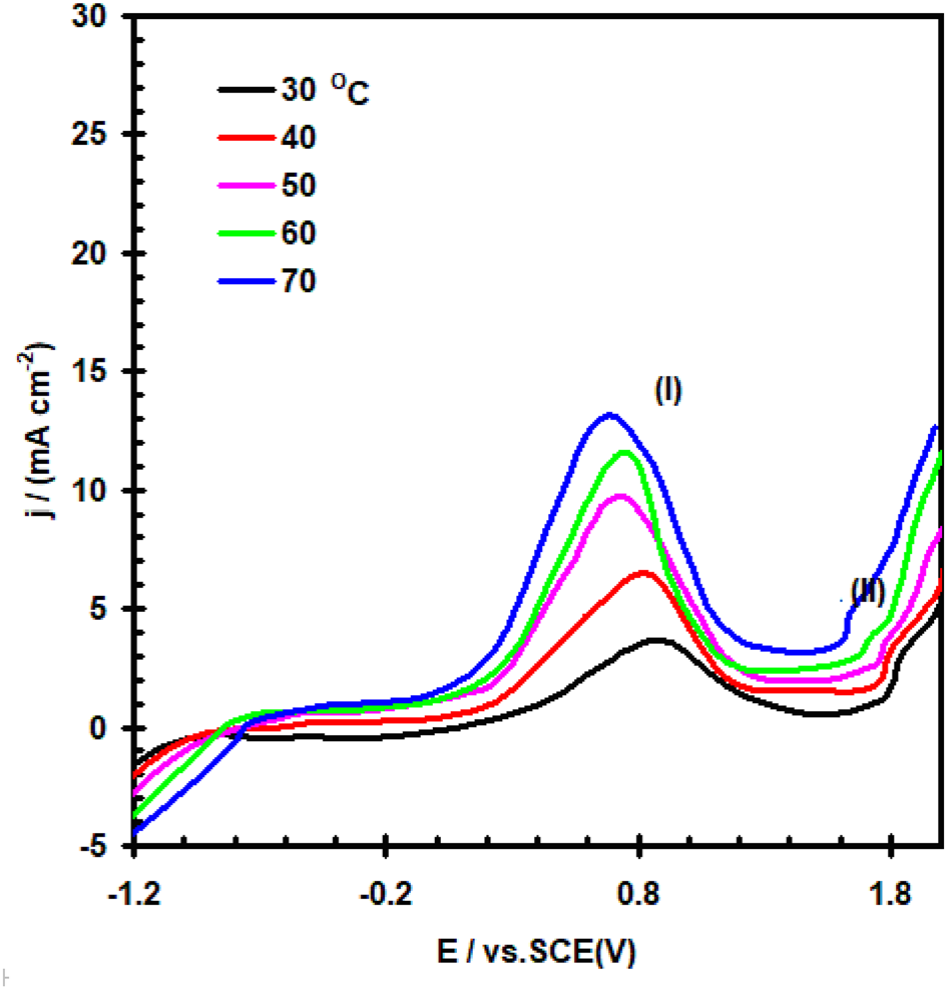
Polarization curve for nickel electrode in 0.4 M of Na_2_SO_4_ solution with scan rate of 20 mVs^–1^ at different temperatures.

Figure 4 shows the effect of solution temperature on the potentiodynamic polarization response of nickel in 0.4 M Na_2_SO_4_ solution at scan rate of 20 mVs^–1^. The data clearly show that, the rise of temperature increases the value of I_PA1_ and slightly shifts its peak potential towords more negative values. This is results can be interpreted on the bases of the fact that, at high temperature, porous oxide film is then, soft, and non-protective. This is because, the solubility of the oxide film by the electrolyte increase with increasing temperature, thus enhancing the anodic dissolution processes^24^. Rise in temperature in temperature also accelerates the transport of reagent and reaction products to and from electrode surface^25^.

**Figure 5:**
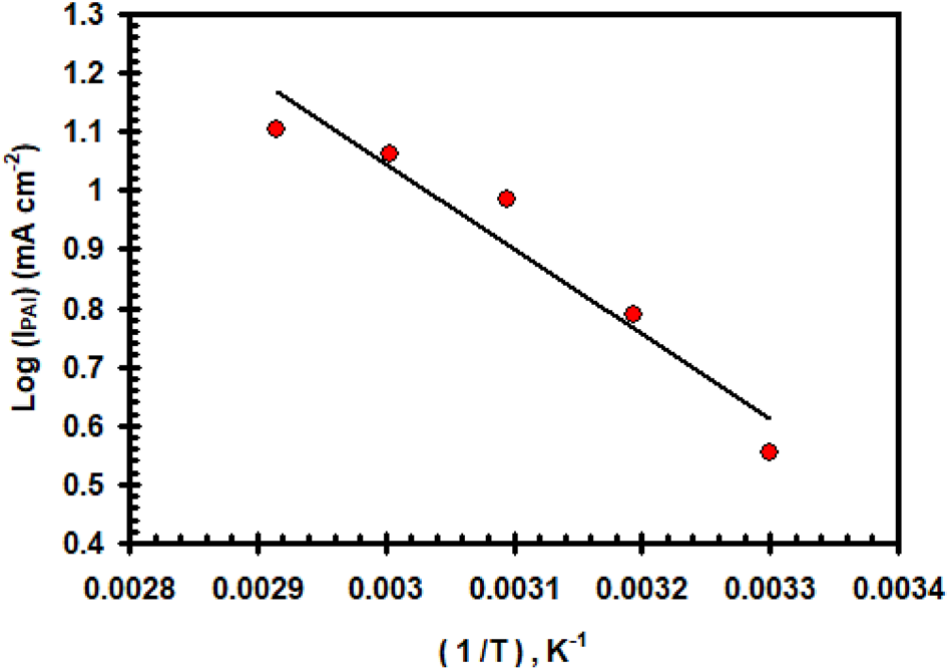
Relation between log (I_PAI_) vs. (1/T) for nickel electrode in 0.4 M of Na_2_SO_4_ solution with scan rate of 20 mVs^–1^.

In Fig. 5, the values of log I_PA1_ are displayed as variables of (1/T)(K) (Arrhenius graph) for several temperatures. Based on the slope of that Arrhenius plotting, the apparent activation energy E_a_ for anodic reaction was determined. The data provide a value for the apparent activation energy, E_a_ = 33.25 kJ mol^–1^.

### Effect ClO_4_^–^ ions on the electrochmeical behavior of nickel

**Figure 6:**
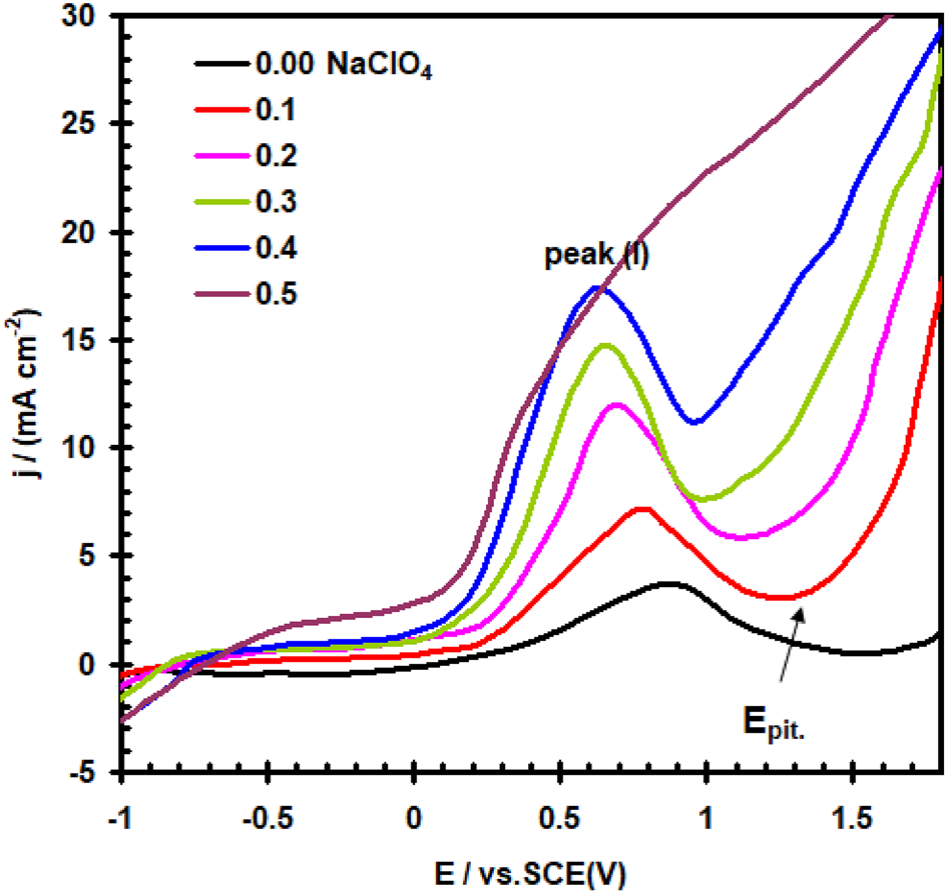
Effect of addition ClO_4_^–^ ion on the potentiodynamic polarization curve for nickel electrode in 0.4 M of Na_2_SO_4_ solution with scan rate of 20 mVs^–1^ at 30 °C.

**Figure 7:**
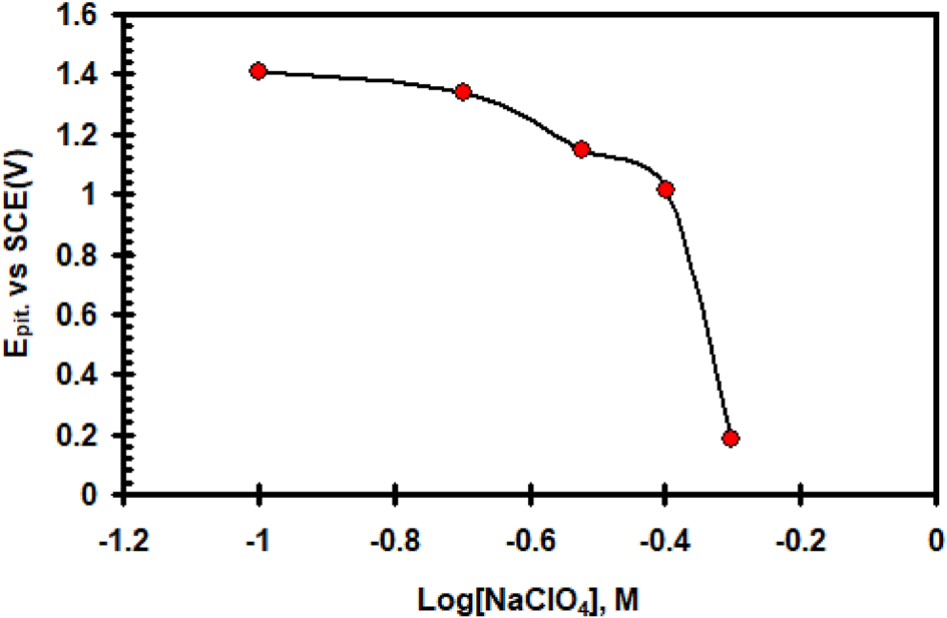
Relation between (E_pit_) vs. log [ClO_4_^–^] for nickel electrode in 0.4 M of Na_2_SO_4_ solution with scan rate of 20 mVs^–1^ at 30 °C.

It was investigated how different NaClO_4_ addition concentrations (0.1–0.5 M) affected the potentiodynamic, E/I, polarization of nickel in 0.4 M Na_2_SO_4_ at 30 °C and 20 mVs^–1^ scan rate (see Fig. 6). The data shows that, the progressive additions of NaClO_4_ cause an increase in the peak current density (I_PA_) of the anodic peaks and shifts its potential to more negative. These findings imply that perchlorate ions disrupt the oxidation operations of nickel electrodes. Competing with SO_4_^2–^ ions for adsorption on the bare metal surface, ClO_4_^–^ ions can take part in electrode dissolution processes directly^26^. However, the ClO_4_^-^ in the sulphate liquid increases I_pass_ and has a tendency to dissolve the passive layer. The passive current goes up abruptly and strongly at a specific critical potential (E_pit_), signifying the breakdown of the passive barrier and the beginning and progression of the pitting. As the amount of ClO_4_^-^ gets higher, the pitting potential (E_pit_) moves to an increasingly negative (active) location. Figure 7 data demonstrates that, in accordance with Eq. (5), E_pit_ is in a linear manner linked to log [ClO_4_^–^].

5 $${\text{E}}_{{{\text{pit}}}} = {\text{ x}} - {\text{y log}}\left( {{\text{ClO}}_{{4}}^{ - } } \right),$$

where x and y are constants and depends on the condations and nature of the electrode.

The adsorption of ClO_4_^–^ on the oxide wrapped layer, specifically at its weakness points and flaws, can be attributed to the breakdown of the definitive passive barrier and the beginning of the pitting corrosion. Pit growth begins earlier when ClO_4_^-^ concentration increases due to its mobility and acidity increases as a result of metal cation hydrolysis within pits^27^.

**Figure 8:**
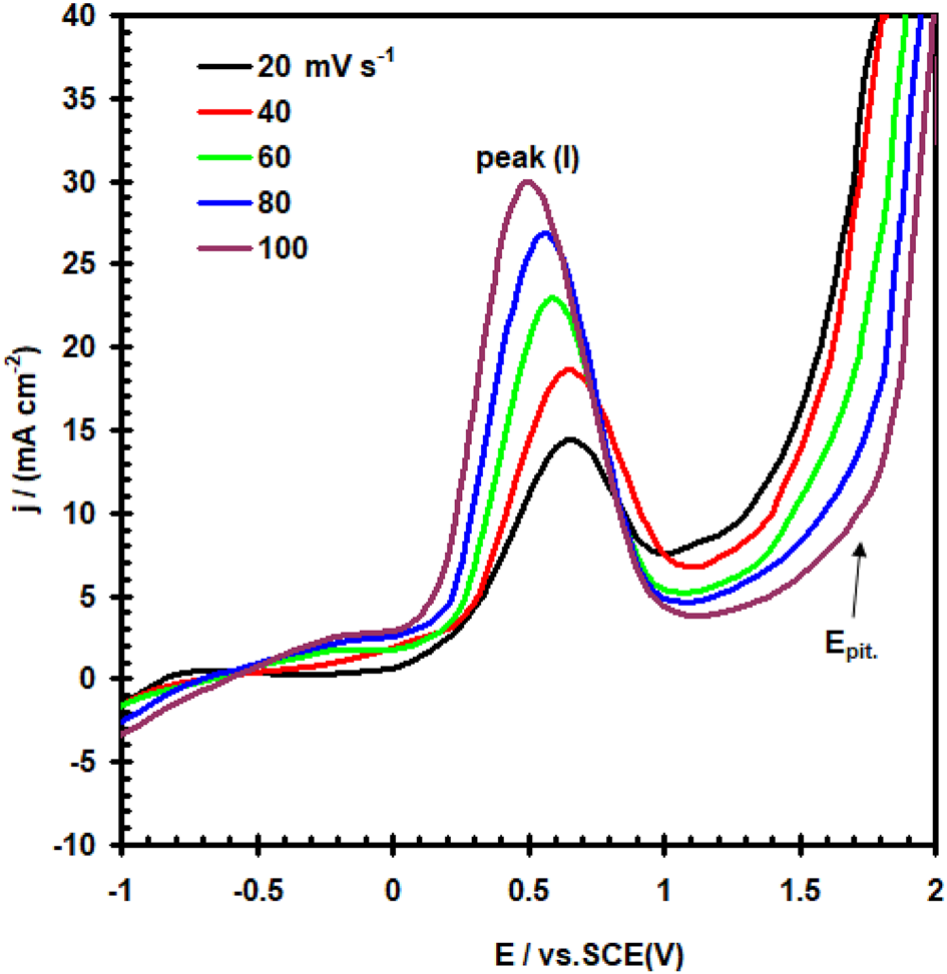
Potentiodynamic polarization curve for nickel electrode in (0.4 M Na_2_SO_4_ + 0.3 M ClO_4_^–^) solution at 30 °C at different scan rate.

In Fig. 8, the significance of the scan rate (υ) regarding the E/I relation of a nickel electrode in a 0.4 M solution of Na_2_SO_4_ containing 0.3 M NaClO_4_ are shown. The anodic peak current is enhanced and has a more negative peak potential as υ increases. Furthermore, the increase in υ shifts E_pit_ towards a more positive value. Incubation time is used to clarify this attitudes^28^. Once the scan has become high, pitting beginning takes place at higher positive potentials, which relate to a short enough incubation period—that is, the period of time required to penetrate the passive layering system.

**Figure 9:**
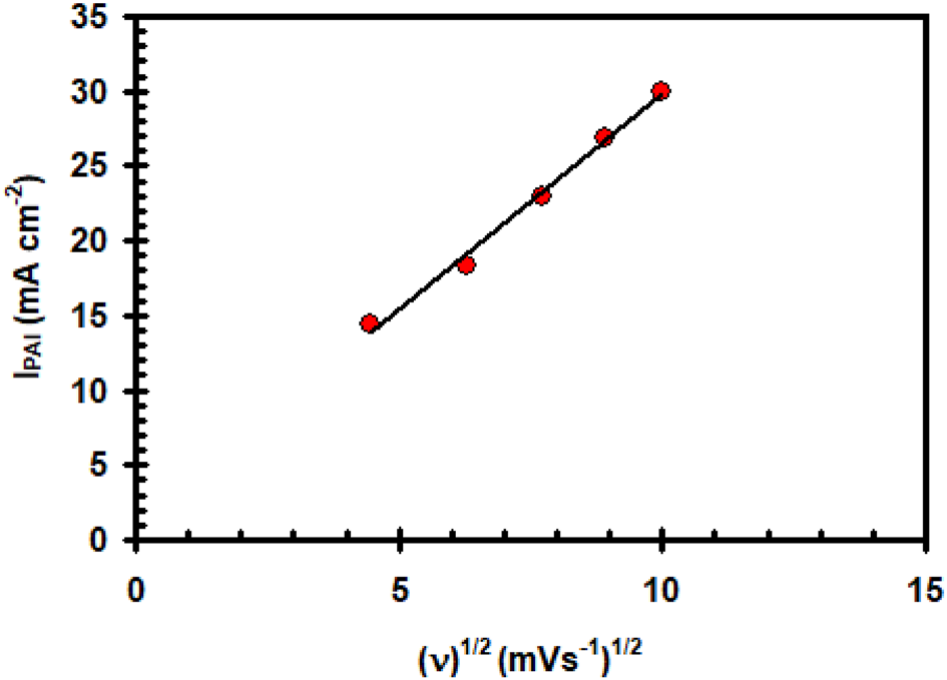
Relation between (I_PAI_) vs. the square root of scan rate for nickel electrode in (0.4 M Na_2_SO_4_ + 0.3 M ClO_4_^–^) solution at 30 °C.

Plotting I_PA_ vs. υ^1/2^ yielded a linear trend depicted in Fig. 9.The resulting straightaway fails to pass through the origin. In theory, the plotting I_PA_ vs. υ^1/2^ shows that diffusion is partially controlling the anodic dissolution operations on nickel in the existence of ClO_4_^–^ anion^29^.

### Effect of added inorganic anions on pitting corrosion

**Figure 10:**
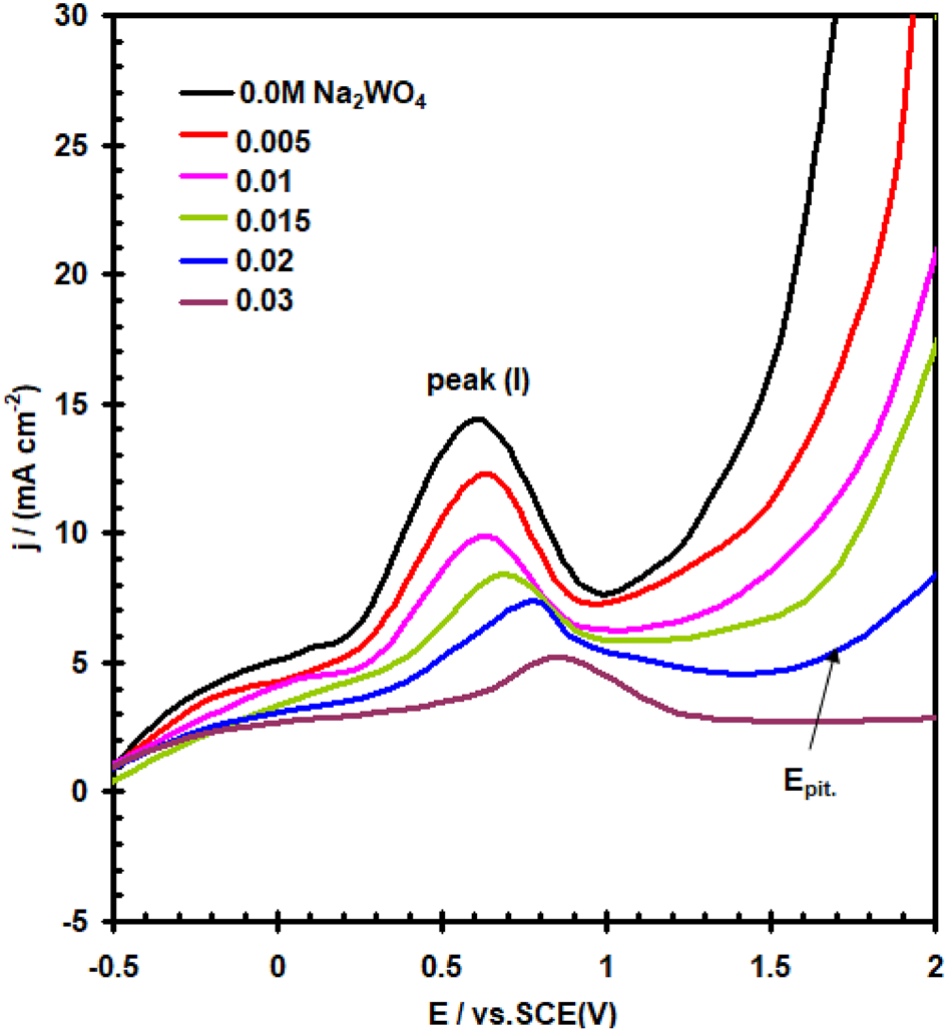
Effect of addition WO_4_^2–^ ions on the potentiodynamic polarization curve for nickel electrode in (0.4 M Na_2_SO_4_ + 0.3 M ClO_4_^–^) solution with scan rate of 20 mVs^–1^ at 30 °C.

**Figure 11:**
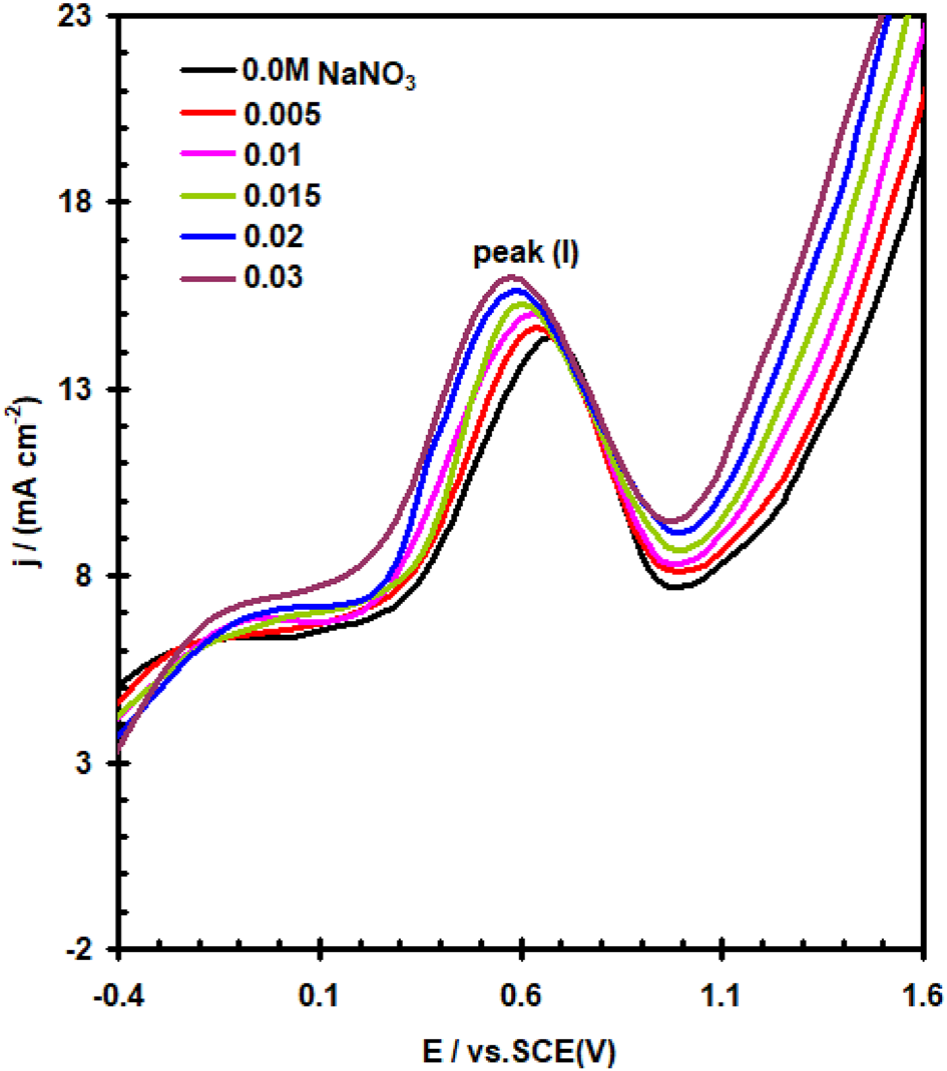
Effect of addition NO_3_^–^ ions on the potentiodynamic polarization curve for nickel electrode in (0.4 M Na_2_SO_4_ + 0.3 M ClO_4_^–^) solution with scan rate of 20 mVs^–1^ at 30 °C.

**Figure 12:**
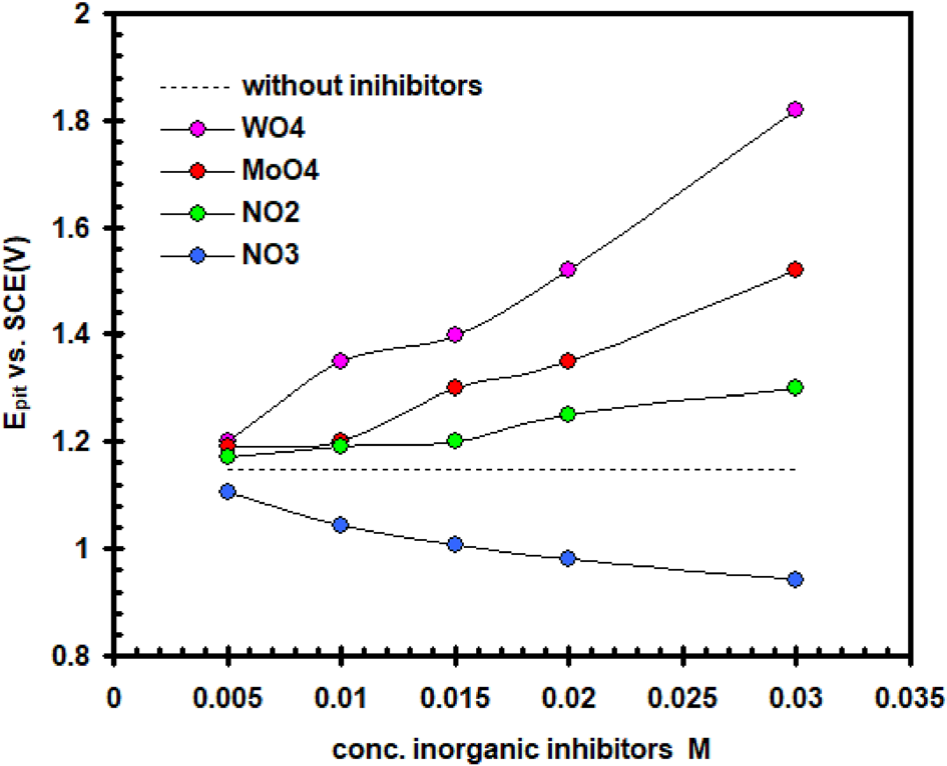
Relationship between (E_pit_) and inorganic inhibitors concentrations for nickel in a solution containing 0.4 M Na_2_SO_4_ and 0.3 M ClO_4_^–^.

The impact of WO_4_^2–^, MoO_4_^2–^, NO_2_^–^ and NO_3_^–^ ions as corrosion-inhibiting agents has been investigated with the goal to learn more concerning the particular function that certain inorganic inhibitors serve on the preventing corrosion mechanisms taking place between the interface between the pure nickel electrode and the electrolyte. E/I curves for nickel in a solution of (0.4 M NaSO_4_ + 0.3 M NaClO_4_) at 30 °C are shown in Figs. 10 and 11, which show the effects when adding different amounts of WO_4_^2–^ and NO_3_^–^ anions in turn. Figure 12 depicts the dependent relationship of E_pit_ on logarithmic inorganic salts concentration.

Analyses of data in Figs. 10, 11, 12 show that, based on the kind and amount of the inhibitor, the existence of all of these anions (except NO_3_^-^), hinders the overall corrosion of nickel to a particular extent. The decline in I_PA_ and the positive departure of the E_pit_ demonstrate this clearly (see Tables 1 and 2). It is evident that these agents have a decreasing effect on nickel’s active dissolution in the following order: WO_4_^2–^ > MoO_4_^2–^ > NO_2_^–^. The adsorption competition of the inhibitor SO_4_^2–^ and ClO_4_^–^ ions on the the outermost layer of the electrode is thought to hinder corrosion. Adsorption and founding of SO_4_^2–^ and ClO_4_^–^ are hindered by the adsorption inhibitor ions. As a result, the SO_4_^2–^ and ClO_4_^–^ anions’surface coverage diminished by the adsorbed inhibitor ions, and the total number of areas of defect at the film’s surface that the anions could enter was also decreased. The difference of the molar polarisation of these ions, which is in the order WO_4_^2–^ > MoO_4_^2–^ > NO_2_^–^, may be responsible for the different anions' inhibitory properties^30^. Because anion molar polarisation is believed to be entirely correlated to ions adsorption^31^, the inhibitory impact should improve with enhancing ions adsorption over the electrode. Surface active ions with higher defarmability are likely to adsorb onto the nickel surface, reducing the actual area that can be used by redox ions for reaction. Figure 11 data show that the NO_3_^–^ anion promotes the I_PAI_ and moves the E_pit._ in a negative way. These impacts may be clarified by the NO_3_^–^ anion’s lower polarizability value with respect to the severe SO_4_^2–^ and ClO_4_^–^ ions^32^.

**Table 1:** The pitting potential (E_pit_) for nickel electrode in (0.4 M Na_2_SO_4_ + 0.3 M ClO_4_^–^) solution in the absence and presence of amounts of WO_4_^2–^, MoO_4_^2–^, NO_2_^–^ and NO_3_^–^ anions.

Inorganic inhibitors conc. M	Blank solution (0.4 M Na_2_SO_4_ + 0.3 M ClO_4_^–^) E_pit_ V(SCE)	E_pit_ V(SCE)			
		WO_4_^2–^	MoO_4_^2–^	NO_2_^–^	NO_3_^–^
0	1.15	–	–	–	–
0.005	–	1.20	1.19	1.17	1.11
0.01	–	1.35	1.20	1.19	1.04
0.015	–	1.40	1.30	1.20	1.01
0.02	–	1.52	1.35	1.25	0.98
0.03	–	1.82	1.52	1.30	0.94

**Table 2:** The anodic peak current density (I_PA1_) for nickel electrode in (0.4 M Na_2_SO_4_ + 0.3 M ClO_4_^–^) solution in the absence and presence of amounts of WO_4_^2–^, MoO_4_^2–^, NO_2_^–^ and NO_3_^–^ anions.

Inorganic inhibitors conc. M	Blank solution (0.4 M Na_2_SO_4_ + 0.3 M ClO_4_^–^) I_PAI_ mA cm^–2^	I_PAI_ mA cm^–2^			
		WO_4_^2–^	MoO_4_^2–^	NO_2_^–^	NO_3_^–^
0	14.4	–	–	–	–
0.005	–	12.3	13.11	13.51	14.66
0.01	–	9.89	11.82	12.58	15.00
0.015	–	8.39	9.53	11.33	15.25
0.02	–	7.36	8.17	10.89	15.58
0.03	–	5.21	7.24	9.94	16.00

The amount of inorganic salts needed to produce a significant positive shift in E_pit_ (as indicated inhibition) improves in the following sequence: NO_2_^–^ > MoO_4_^2–^ > WO_4_^2–^ (see Fig. 12). Because of their particular adsorbility, these inhibitors are able to remove ClO_4_^–^ ion from the locations where it more readily enters the passive coating, which improves pitting resistance against corrosion^33,34^.

Furthermore, the reduced modes of these inhibitors (WO_2_ and MoO_2_), for example, transform into component of the passivating oxide and have a tendency to block its pores and weaknesses getting more effectively shielding capabilities. on the additional one, nitrite ions had been found to be reduced to ammonia in which oxygen that remained on the surface triggered the oxide reaction^35^. Figure 12 data demonstrate that the incorporation of NO_3_^-^ ions moves E_pit_ to a more negative direction, demonstrating that NO_3_^-^ ions encourage pitting corrosion. The finding could be explained by the relatively low polarizability of NO_3_^–^ ions^36^ in comparison to other ions.

The inhibition corrosion efficiency (*IE*%) of various inorganic ions was estimated as follows^37^:

6 $${\text{IE}}\,\% = \frac{{{\text{I}}_{{{\text{PA}}(0)}} - {\text{I}}_{{{\text{PA}}}} }}{{{\text{I}}_{{{\text{PA}}(0)}} }} \times 100,$$

where I_PA(0)_ is the anodic current density in the absence inorganic ions. The inhibitory efficiency improves as the concentration of inorganic ions increases (see Table 3). The sequence of increase in the inhibitors' inhibitory effectiveness is WO_4_^2–^ > MoO_4_^2–^ > NO_2_^–^.

**Table 3:** The corrosion inhibition efficiency of inorganic inhibitors for nickel in (0.4 M Na_2_SO_4_ + 0.3 M ClO_4_^–^) solution.

Inorganic inhibitors conc. M	*IE*%		
	WO_4_^2–^	MoO_4_^2–^	NO_2_^–^
0.005	14.5	8.9	6.1
0.01	31.3	17.9	12.6
0.015	41.7	33.8	21.3
0.02	48.8	43.2	24.3
0.03	63.8	49.7	39.9

### Surface studies

The surface morphology caused by immersion in the corrosive solution (0.4 M Na_2_SO_4_ + 0.3 M NaClO_4_) in the presence and absence of the compounds under consideration (ClO_4_^–^, WO_4_^2–^, MoO_4_^2–^, NO_2_^–^ and NO_3_^–^ ions, 0.03 M) were compared using the SEM methodology on nickel specimens.

**Figure 13:**
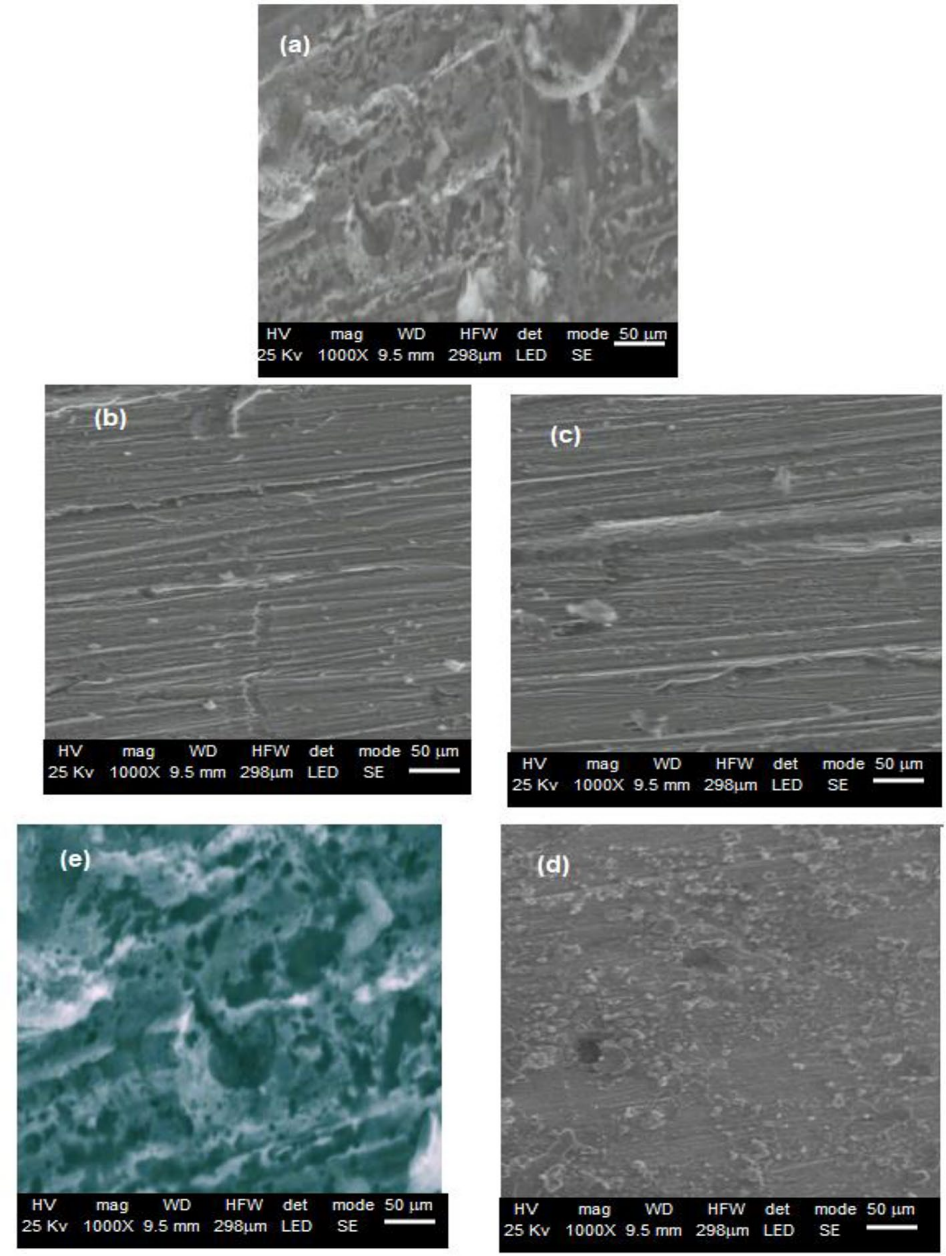
SEM images of Nickel samples in corrosive medium (0.4 M NaSO_4_ + 0.3 M NaClO_4_) (image (**a**)) and in the presence of the inhibitors, WO_4_^2–^ (image (**b**)), MoO_4_^2–^ (image (**c**)), NO_2_^–^ (image (**d**)) and NO_3_^–^ (image (**e**)). Immersion time: 120 h. Temperature: 30 °C.

The nickel specimens were subjected to (0.4 M Na_2_SO_4_ + 0.3 M NaClO_4_) for 120 h around 30 degrees Celsius, and then they were taken from the solution and allowed to sit for an additional hour to dry before examination. According to Fig. 13a depicts the image of the untreated sample in the corrosive solution (0.4 M Na_2_SO_4_ + 0.3 M NaClO_4_). In the absence of inhibitors, the large damaged regions, evident deteriorations, and pitting holes are visible.

In contrast, the microscope images of the tested nickel samples in the presence of the inhibitors, WO_4_^2–^, MoO_4_^2–^, NO_2_^–^ and NO_3_^–^, in Fig. 13b–d, and e, respectively, showed highly effective corrosion inhibition efficiency as evidenced by the disappearance of deterioration areas, the disappearance of localized corrosion areas, and the fit (except NO_3_^–^).

The nickel surface was found to be very corrosive in the presence of NO_3_^-^ions (Fig. 13e). The present SEM results and the prior electrochemical technique results both show that the examined inhibitors (with the exception of NO_3_^–^) have effective corrosion control.

## Conclusion

The current study investigates the anti-corrosion capabilities of various inorganic materials, such as WO_4_^2–^, MoO_4_^2–^, NO_2_^–^ and NO_3_^–^, on nickel in Na_2_SO_4_ solution in the presence of perchlorate ions. Potentiodynamic curves show one dissolution peak (I), definitive passive, and transpassive state (II) regions. The data reveal that the anodic current density increase with increase in Na_2_SO_4_ solution concentration, potential scan rate (υ) and solution temperature. The progressive additions of NaClO_4_ cause an increase in corrosion of nickel electrode and tend to breakdown the passive layer. The presence of the inorganic anions studied (Except NO_3_^–^) inhibits both anodic dissolution and pitting corrosion of nickel. The sequence of increase in the inhibitors' inhibitory effectiveness is WO_4_^2–^ > MoO_4_^2–^ > NO_2_^–^.

## Data Availability

The datasets used and/or analysed during the current study available from the corresponding author on reasonable request.
